# Green Cleaning of 3D-Printed Polymeric Products by Micro-/Nano-Bubbles

**DOI:** 10.3390/nano13111804

**Published:** 2023-06-05

**Authors:** Haoxiang Gao, Fenghua Zhang, Kangkang Tang, Xianyu Luo, Ziang Pu, Jiuzhou Zhao, Zhiwei Jiao, Weimin Yang

**Affiliations:** College of Mechanical and Electrical Engineering, Beijing University of Chemical Technology, Beijing 100029, China

**Keywords:** cleaning, 3D printing, micro-/nano-bubbles, ultrasound, porous polymers

## Abstract

3D printing technology has been used to directly produce various actual products, ranging from engines and medicines to toys, especially due to its advantage in producing items of complicated, porous structures, which are inherently difficult to clean. Here, we apply micro-/nano-bubble technology to the removal of oil contaminants from 3D-printed polymeric products. Micro-/nano-bubbles show promise in the enhancement of cleaning performance with or without ultrasound, which is attributed to their large specific surface area enhancing the adhesion sites of contaminants, and their high Zeta potential which attracts contaminant particles. Additionally, bubbles produce tiny jets and shock waves at their rupture, driven by coupled ultrasound, which can remove sticky contaminants from 3D-printed products. As an effective, efficient, and environmentally friendly cleaning method, micro-/nano-bubbles can be used in a range of applications.

## 1. Introduction

3D printing technology can directly print material-deposited objects layer by layer from computer-aided design (CAD) models [1]. In the past, it was often used in mold manufacturing, industrial design, and other fields to manufacture models. However, some companies have started using 3D-printed components as actual components [2], and the technology has shown promise in applications in clinical medicine [3], mechanical products [4,5], clothing [6], furniture [7], etc.

3D printing technology is characterized by its ability to create objects of almost any shape, but micro particles or contaminants such as oil are frequently adsorbed on the inner surfaces of the products. In recent years, researchers have identified several types of micro plastics in blood, breast milk, feces, etc. [8,9,10,11,12], which can enter the body directly through water, food, the respiratory system, the eyes, or open wounds. The accumulation of plastic micro particles in the human body can cause disease or exacerbate discomfort, and the toxic substances they contain or carry can be slowly dislodged and precipitated in the body via the continued fragmentation of the particles or the action of enzymes in the body, causing a variety of health issues. The special porous structure makes the pollutants difficult to remove via traditional cleaning methods. Therefore, a safe and green cleaning technology for 3D-printed products is urgently needed. Micro-/nano-bubble cleaning technology offers a new option to meet the demand.

Micro-/nano-bubbles are bubbles with a diameter of less than 100 μm [13]. With properties such as a high internal gas density [14], a large specific surface area, a spontaneously charged surface, and the ability to generate free radicals [15], they have been extensively used in promoting biological growth [16], improving the soil environment [17,18], wastewater treatment [19], targeted drug delivery [20], and ultrasonography [21]. At present, micro-/nano-bubble technology has shown good cleaning effects on excavated porcelain with hard adherents on the surface [22], fruits and vegetables with microbial contamination on the surface [23,24], oily soil [25], membrane contaminants [26], and grease on metal surfaces [27,28], etc. Here, we study the cleaning effect of water containing micro-/nano-bubbles on the 3D-printed products of polymers.

## 2. Materials and Methods

### 2.1. Materials and Characterization

**Figure 1:**
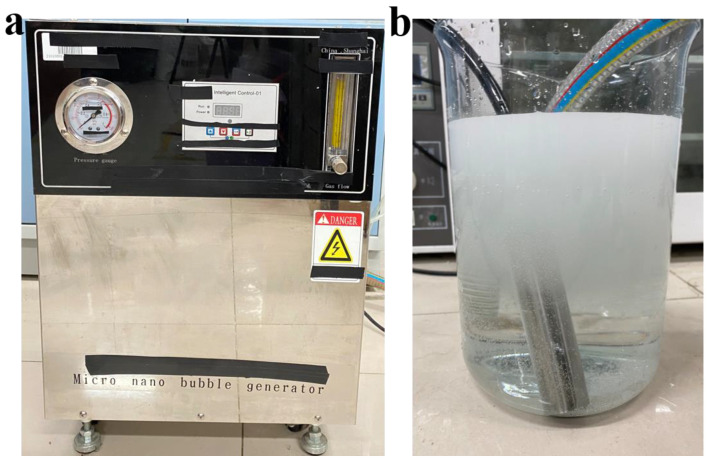
The micro-/nano-bubble generator used (**a**) and a snapshot of the produced micro-/nano-bubbles in DI water (**b**).

**Figure 2:**
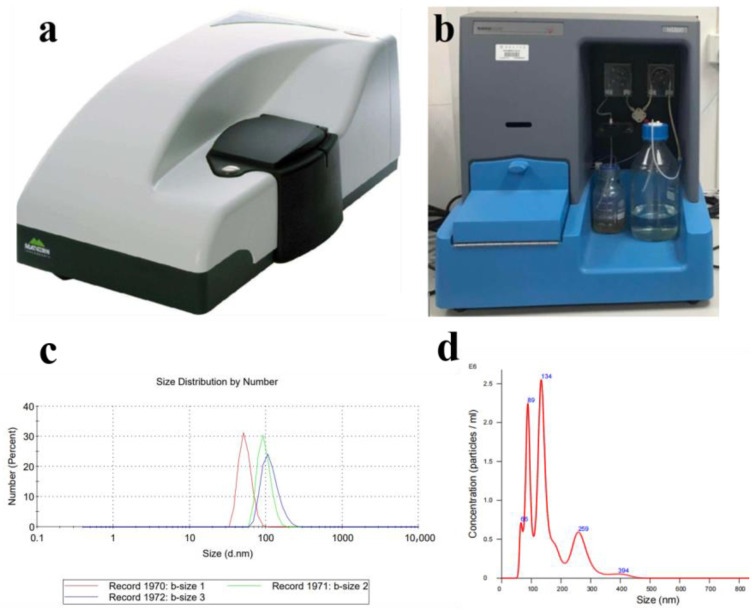
(**a**) Particle size analyzer of Zetasizer (ZS90). (**b**) Nanoparticle tracking analyzer (NTA). Characterization of the nanobubbles by using Zetasizer (**c**) and NTA (**d**).

**Figure 3:**
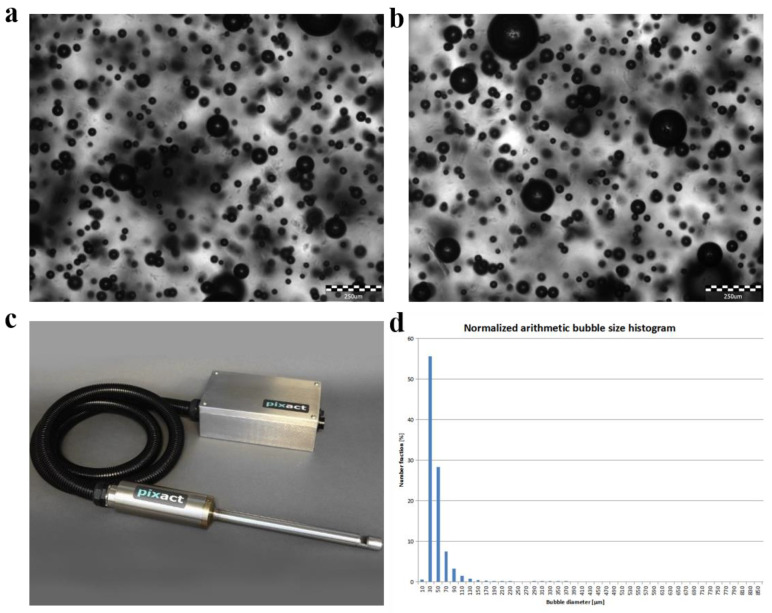
Example snapshots (**a**,**b**) of the microbubbles captured by using a Pixact particle imaging device (**c**). (**d**) The measured size distribution of the microbubbles.

We used deionized (DI) water, air, and a laboratory-made micro-/nano-bubble generator to prepare the bubbly water (Figure 1) by mechanical-shear- and Venturi-type flow. When the generation eased, the microbubbles (white, milky) ascended to the bulk surface due to the buoyancy, and disappeared in about 10 min, with nanobubbles (transparent) left in the water. Samples of the water with nanobubbles were characterized by using Zetasizer (ZS90, Malvern Instruments, Malvern, UK) and nanoparticle tracking analyzer (Nanosight 500, Malvern Instruments, Malvern, UK), as shown in Figure 2. The three measurements performed by using DLS, and the average curve of five measurements performed by using NTA showed that the nanobubbles were within 50–450 nm in diameter, with the concentration of 1.94·10^8^ bubbles in per ml of water. The microbubbles were measured by using a microscopic imaging device (PBM, Pixact, Tampere, Finland), as shown in Figure 3. Plenty of snapshots were recorded and image analysis was performed using the associated software to produce the size distribution of the microbubbles. As shown in Figure 3d, more than 97% of the bubbles captured were less than 100 μm in diameter. The zeta potential of the bubbly water was about −20 mV, measured by using Zetasizer (ZS90, Malvern Instruments, Malvern, UK). The oil used was VG100 (Great wall).

### 2.2. Cleaning Operations

The artificial addition of oil was conducted via immersion for 10 s of several similar 3D-printed products, once together into oil in a glass Petri dish. Then, the contaminated products were put in a clean and covered glass Petri dish for 4 h. After that, the products were taken out, weighed, and recorded as the “weight with oil”, where the residual oil was a relatively stable contaminant to the products. The different pieces of 3D-printed products had slightly different net weights, and carried random amounts of oil with small variations.

**Figure 4:**
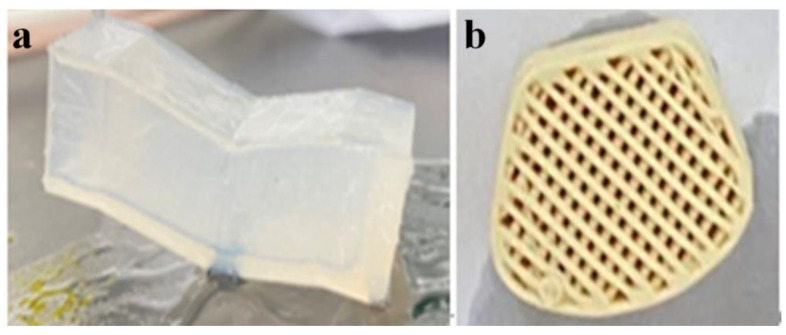
Images of the 3D-printed porous products of rubber (**a**) and plastics (**b**).

We used DI water to soak and agitate the oil-stained, 3D-printed porous rubber (silica gel) or plastic (polypropylene) products (Figure 4) for a while, and dried them in a vacuum drying oven at 60 °C for 2 h to remove the residual water in the pores. The cleaning rate was then analyzed. For each case of cleaning, we carried out four types of experiments of cleaning: DI water, DI water with micro-/nano-bubbles, DI water with ultrasound, and DI water containing micro-/nano-bubbles coupled with ultrasound. The ultrasonic waves were generated by a small ultrasonic cleaner at a frequency of 40 kHz and a power of 35 W. We also compared cleaning by soaking and stirring using an electric stirrer with blades, at a rotating speed of 600 r/s.

### 2.3. Evaluation of Oil Removal Rate

We used a precision electronic balance with an accuracy of 0.01 g to weigh the initial clean product, the oiled 3D-printed product, and the cleaned and dried 3D-printed product, with the measured weight represented by A, B, and C, respectively.

The oil removal rate was then evaluated as η = E/D, where the total weight of additional oil D = B − A, and the weight of oil removed E = B − C. The final masses of the initial products after cleaning with detergents and drying appeared same as the initial values, indicating that the above calculation of the removal rate was correct, excluding the influence of potential corrosion and other contaminants to the measurement and evaluation of the cleaning effect. All the experiments were done in open vessels with a water temperature of 22 °C before the cleaning, and a temperature of 22–28 °C after the various phases of cleaning. The small variation in water temperature may not induce significant changes in the removal rate of oil.

## 3. Results and Discussion

### 3.1. Cleaning Rubber Products

#### 3.1.1. Cleaning by Soaking

**Figure 5:**
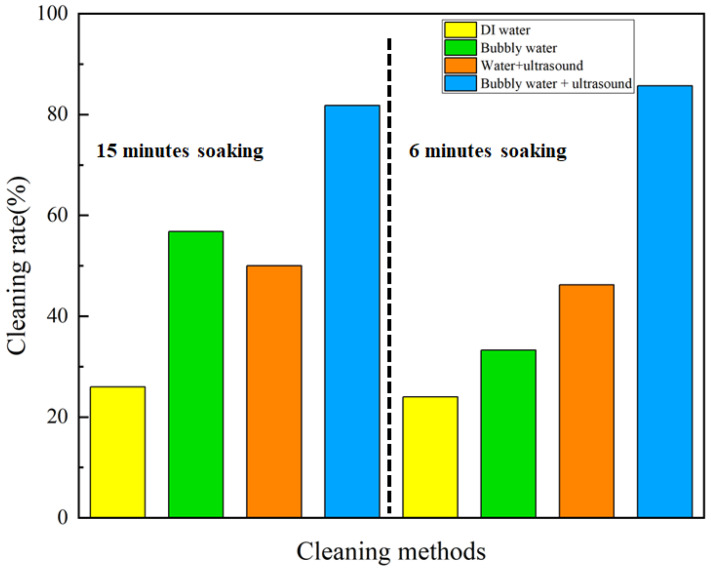
Comparison of the cleaning rate for 3D-printed rubber products by soaking.

We cleaned the 3D-printed, porous rubber products with different periods of soaking, and the cleaning results are shown in Table 1 and Table 2. The removal rates were 26.0%, 56.8%, 50.0%, 81.8% for soaking in DI water, bubbly water, water with ultrasound, and bubbly water with ultrasound, respectively. Taking the case of DI water as the control, the data in Table 1 and the illustrated data in Figure 5 show that, for cleaning 3D-printed, porous rubber products by mere soaking for 15 min, the addition of micro-/nano-bubbles to the DI water increased the oil removal rate by 118.4%, while applying ultrasound increased it by 92.3%, and coupled cleaning of micro-/nano-bubbles and ultrasound achieved the maximum increase of 214.6%.

In the interest of saving energy, we performed the same cleaning experiment again for a shorter time, i.e., soaking for 6 min. The results showed that the addition of micro-/nano-bubbles or ultrasound improved the cleaning rate by 38.8% or 92.5%, respectively, and the coupled micro-/nano-bubbles and ultrasound improved it by 257.1%, compared with DI water.

The results show that applying either micro-/nano-bubbles or ultrasound is effective in increasing the cleaning rate of 3D-printed rubber products. Moreover, the combination of micro-/nano-bubbles and ultrasound achieved the best cleaning effect. Specific surface area of a bubble is inversely proportional to its radius; therefore, micro-/nano-bubbles have a large specific surface area, which increases the probability of attachment with surface contaminants [29]. In addition, the surfaces of micro-/nano-bubbles tend to carry charges, as evidenced by the high Zeta potential [30], becoming attractive to small particles. Therefore, micro-/nano-bubbles can effectively capture pollutant particles in the pores of 3D-printed products. Furthermore, ultrasound can break some of the micro-/nano-bubbles, forming a micro-/nano- jet impact on the nearby solid surface, which is highly conducive to the removal of contaminants from a product’s surface.

#### 3.1.2. Cleaning with Stirring

We performed the cleaning experiments with mechanical stirring for various periods of time, and the results are shown in Table 3 and Table 4.

**Figure 6:**
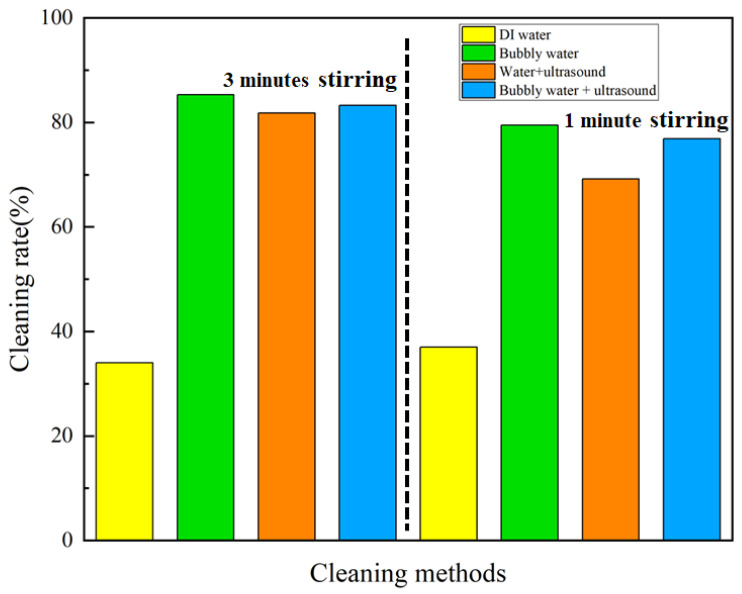
Comparison of the cleaning rate for 3D-printed rubber products via stirring.

In cleaning with stirring for 3 min, the results show that the addition of micro-/nano-bubbles or ultrasound, and the coupled micro-/nano-bubbles and ultrasound improved the cleaning rate by 150.9%, 140.6%, and 145%, respectively, compared with DI water. A shorter duration of cleaning with stirring for 1 min achieved an increase in the cleaning rate by 114.9%, 87.0%, and 107.8%, respectively, as illustrated in Figure 6. The results show that applying either micro-/nano-bubbles or ultrasound, or the coupled micro-/nano-bubbles and ultrasound is effective in increasing the cleaning rate of 3D-printed rubber products. However, the micro-/nano-bubbles achieved the best cleaning effect. This indicates that when dynamic cleaning methods such as stirring are applied, the flush of water along the product’s surface evidently enhances the cleaning effect, and the addition of micro-/nano-bubbles offers plenty of additional surfaces to stick and carry the oil droplets away from the products.

### 3.2. Cleaning Plastic Products

#### 3.2.1. Cleaning by Soaking

We also cleaned 3D-printed, porous plastic products by soaking of different periods of time. The results are shown in Table 5 and Table 6.

**Figure 7:**
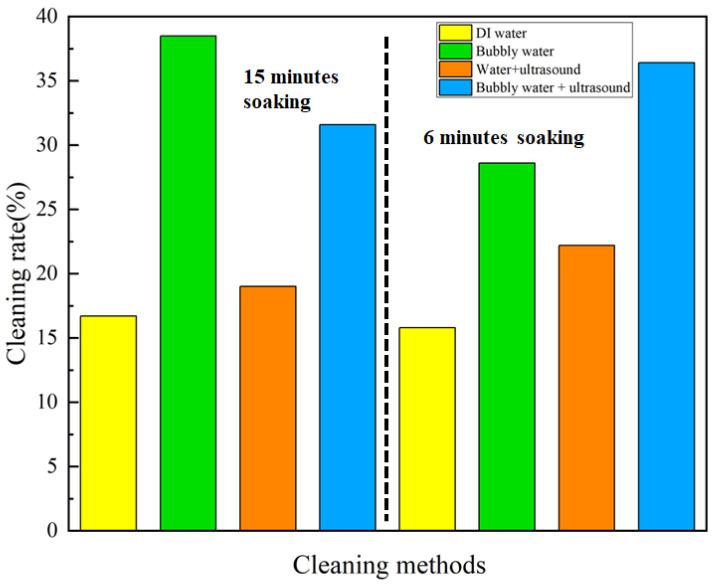
Comparison of the cleaning rate for 3D-printed plastic products via soaking.

As illustrated in Figure 7, in the case of cleaning via soaking for 15 min, the addition of micro-/nano-bubbles or ultrasound, and the coupled micro-/nano-bubbles and ultrasound, improved the cleaning rate by 130.5%, 13.8%, and 89.2%, respectively, relative to DI water. In the case of cleaning via soaking for 6 min, the addition of micro-/nano-bubbles or ultrasound, and the coupled micro-/nano-bubbles and ultrasound, improved the cleaning rate by 81.0%, 40.5%, and 130.4%, respectively.

#### 3.2.2. Cleaning with Stirring

**Figure 8:**
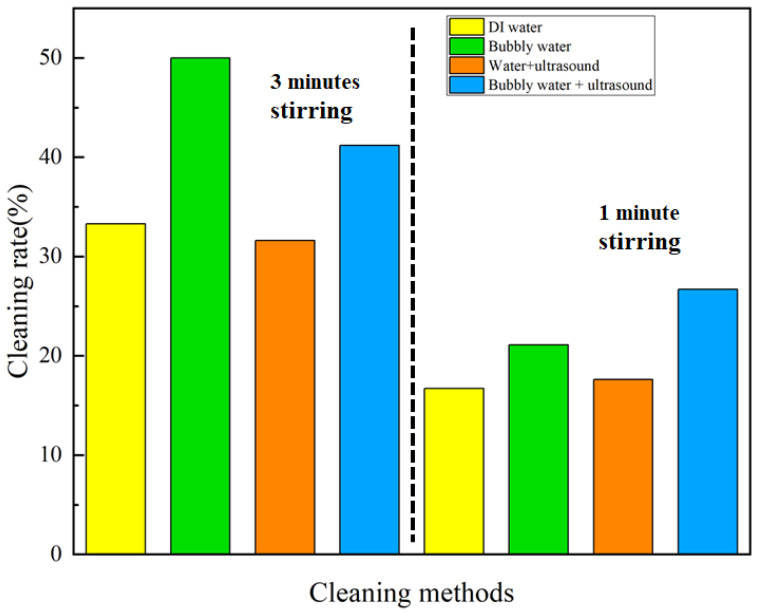
Comparison of the cleaning rate for 3D-printed plastic products via stirring.

Experiments were also performed in cleaning 3D-printed plastic products with mechanical stirring for various durations, and the results are shown in Table 7 and Table 8. As illustrated in Figure 8, the results of cleaning with stirring for 3 min show that the addition of micro-/nano-bubbles or ultrasound, and the coupled micro-/nano-bubbles and ultrasound increased the cleaning rate by 50.2%, −5.1%, and 23.7%, respectively, compared with DI water. A shorter duration of cleaning with stirring for 1 min achieved an increase in the cleaning rate of 26.3%, 5.4%, and 60.0%, respectively.

For the cleaning of 3D-printed plastic products, the results of both soaking and stirring show that applying either micro-/nano-bubbles or ultrasound, or the coupled micro-/nano-bubbles and ultrasound is effective in increasing the cleaning rate. Although it is apparent that the micro-/nano-bubbles, or bubbles + ultrasound achieved better cleaning than the other two methods. However, the relative advantage between micro-/nano-bubbles and bubbles + ultrasound is not definite. This may be attributed to the fact that ultrasound can produce cavitation bubbles [31,32], but can also kill initial gas bubbles, producing a degassing effect. After the oil-droplet-borne bubbles rupture via ultrasound, the detached oil droplets are likely to stick again to the plastic surfaces [33,34,35].

We speculate that the difference was due to the different structures of the rubber and the plastic products. As shown in Figure 4, the latter had more complex structures and inner pores. A longer stirring duration could flush away the oil in deep holes and corners. This may be further studied in the future with the cleaning of the same structure of different materials, or vice versa.

After the cleaning experiments, the oily products were further carefully cleaned with detergents, then dried in a vacuum drying oven at 60 °C for 2 h, and finally weighed. The final weights of the various pieces of products were almost the same as the initial values, indicating that no notable corrosion by the oil, nor apparent release of the rubber or plastic from the products had occurred during the experiments. The corrosion may become apparent with longer durations of oil contamination.

## 4. Conclusions

In summary, we prepared a high concentration of approximately 0.2 billion micro-/nano-bubbles per ml of DI water with microbubbles of less than 100 μm in diameter and nanobubbles mainly within 50–450 nm in diameter. We subsequently explored their cleaning effect on 3D-printed, porous rubber and plastic products in various cases. The addition of micro-/nano-bubbles to the water significantly increased the cleaning effect of 3D-printed, porous rubber and plastic products. Although ultrasound also showed a good cleaning effect relative to pure water, its performance was further enhanced when coupled with micro-/nano-bubbles. The enhancement was mainly attributed to the inherent properties of the micro-/nano-bubbles, including a large specific surface area which raises the probability of adhering to surface contaminants, and a high Zeta potential which is attractive to small contaminant particles. In addition, when coupled with ultrasound, micro-/nano-bubbles may break, forming micro-/nano- jets and shock waves, which is highly effective and efficient in the removal of contaminants from the complex inner pores of 3D-printed products.

## Figures and Tables

**Table 1:** Cleaning of rubber products by soaking for 15 min.

	Net Weight (g)	Weight with Oil (g)	Weight after Cleaning (g)	Weight of Oil Carried (g)	Weight of Oil Removed (g)	Removal Rate of Oil (%)
Water	4.31	4.81	4.68	0.50	0.13	26.0
Bubbly water	4.31	5.49	4.82	1.18	0.67	56.8
Water + ultrasound	4.16	4.40	4.28	0.24	0.12	50.0
Bubbly water + ultrasound	4.00	4.22	4.04	0.22	0.18	81.8

**Table 2:** Cleaning of rubber products by soaking for 6 min.

	Net Weight (g)	Weight with Oil (g)	Weight after Cleaning (g)	Weight of Oil Carried (g)	Weight of Oil Removed (g)	Removal Rate of Oil (%)
Water	4.25	4.75	4.63	0.5	0.12	24.0
Bubbly water	4.39	4.69	4.59	0.30	0.10	33.3
Water + ultrasound	4.16	4.42	4.30	0.26	0.12	46.2
Bubbly water + ultrasound	4.00	4.28	4.04	0.28	0.24	85.7

**Table 3:** Cleaning of rubber products via stirring for 3 min.

	Net Weight (g)	Weight with Oil (g)	Weight after Cleaning (g)	Weight of Oil Carried (g)	Weight of Oil Removed (g)	Removal Rate of Oil (%)
Water	4.35	4.88	4.70	0.53	0.18	34.0
Bubbly water	4.52	4.86	4.57	0.34	0.29	85.3
Water + ultrasound	4.30	4.52	4.34	0.22	0.18	81.8
Bubbly water + ultrasound	4.30	4.54	4.34	0.24	0.20	83.3

**Table 4:** Cleaning of rubber products via stirring for 1 min.

	Net Weight (g)	Weight with Oil (g)	Weight after Cleaning (g)	Weight of Oil Carried (g)	Weight of Oil Removed (g)	Removal Rate of Oil (%)
Water	4.17	4.63	4.46	0.46	0.17	37.0
Bubbly water	4.36	4.75	4.44	0.39	0.31	79.5
Water + ultrasound	4.30	4.56	4.38	0.26	0.18	69.2
Bubbly water + ultrasound	4.36	4.62	4.42	0.26	0.20	76.9

**Table 5:** Cleaning of plastic products by soaking for 15 min.

	Net Weight (g)	Weight with Oil (g)	Weight after Cleaning (g)	Weight of Oil Carried (g)	Weight of Oil Removed (g)	Removal Rate of Oil (%)
Water	0.41	0.59	0.56	0.18	0.03	16.7
Bubbly water	0.41	0.67	0.57	0.26	0.10	38.5
Water + ultrasound	0.39	0.60	0.56	0.21	0.04	19.0
Bubbly water + ultrasound	0.44	0.63	0.57	0.19	0.06	31.6

**Table 6:** Cleaning of plastic products by soaking for 6 min.

	Net Weight (g)	Weight with Oil (g)	Weight after Cleaning (g)	Weight of Oil Carried (g)	Weight of Oil Removed (g)	Removal Rate of Oil (%)
Water	0.46	0.65	0.62	0.19	0.03	15.8
Bubbly water	0.43	0.64	0.58	0.21	0.06	28.6
Water + ultrasound	0.51	0.69	0.65	0.18	0.04	22.2
Bubbly water + ultrasound	0.40	0.62	0.54	0.22	0.08	36.4

**Table 7:** Cleaning of plastic products via stirring for 3 min.

	Net Weight (g)	Weight with Oil (g)	Weight after Cleaning (g)	Weight of Oil Carried (g)	Weight of Oil Removed (g)	Removal Rate of Oil (%)
Water	0.50	0.65	0.60	0.15	0.05	33.3
Bubbly water	0.53	0.69	0.61	0.16	0.08	50.0
Water + ultrasound	0.40	0.59	0.53	0.19	0.06	31.6
Bubbly water + ultrasound	0.42	0.66	0.56	0.24	0.10	41.2

**Table 8:** Cleaning of plastic products via stirring for 1 min.

	Net Weight (g)	Weight with Oil (g)	Weight after Cleaning (g)	Weight of Oil Carried (g)	Weight of Oil Removed (g)	Removal Rate of Oil (%)
Water	0.44	0.62	0.59	0.18	0.03	16.7
Bubbly water	0.40	0.59	0.55	0.19	0.04	21.1
Water + ultrasound	0.51	0.68	0.65	0.17	0.03	17.6
Bubbly water + ultrasound	0.38	0.53	0.49	0.15	0.04	26.7

## Data Availability

Not applicable.
